# Connexin43 enhances the expression of osteoarthritis-associated genes in synovial fibroblasts in culture

**DOI:** 10.1186/1471-2474-15-425

**Published:** 2014-12-11

**Authors:** Aditi Gupta, Corinne Niger, Atum M Buo, Eric R Eidelman, Richard J Chen, Joseph P Stains

**Affiliations:** Department of Orthopaedics, University of Maryland School of Medicine, 100 Penn Street, Allied Health Building, Room 540E, Baltimore, MD 21201 USA

**Keywords:** Synovial fibroblasts, Connexin, Gap junction, Signal transduction, NFκB, RelA, Osteoarthritis, Cytokines

## Abstract

**Background:**

Recent work has shown that the gap junction protein connexin43 (Cx43) is upregulated in cells of the joint during osteoarthritis (OA). Here we examined if the OA-associated increase in Cx43 expression impacts the function of synovial fibroblasts by contributing to the production of catabolic and inflammatory factors that exacerbate joint destruction in arthritic disease.

**Methods:**

Using rabbit and human synovial fibroblast cell lines, we examined the effects of Cx43 overexpression and Cx43 siRNA-mediated knockdown on the gene expression of OA-associated matrix metalloproteinases (*MMP1* and *MMP13*), aggrecanases (*ADAMTS4* and *ADAMTS5*), and inflammatory factors (*IL1*, *IL6 and PTGS2*) by quantitative real time RT-PCR. We examined collagenase activity in conditioned media of cultured synovial cells following Cx43 overexpression. Lastly, we assessed the interplay between Cx43 and the NFκB cascade by western blotting and gene expression studies.

**Results:**

Increasing Cx43 expression enhanced the gene expression of *MMP1*, *MMP13*, *ADAMTS4*, *ADAMTS5*, *IL1*, *IL6 and PTGS2* and increased the secretion of collagenases into conditioned media of cultured synovial fibroblasts. Conversely, knockdown of Cx43 decreased expression of many of these catabolic and inflammatory genes. Modulation of Cx43 expression altered the phosphorylation of the NFκB subunit, p65, and inhibition of NFκB with chemical inhibitors blocked the effects of increased Cx43 expression on the mRNA levels of a subset of these catabolic and inflammatory genes.

**Conclusions:**

Increasing or decreasing Cx43 expression alone was sufficient to alter the levels of catabolic and inflammatory genes expressed by synovial cells. The NFκB cascade mediated the effect of Cx43 on the expression of a subset of these OA-associated genes. As such, Cx43 may be involved in joint pathology during OA, and targeting Cx43 expression or function may be a viable therapeutic strategy to attenuate the catabolic and inflammatory environment of the joint during OA.

**Electronic supplementary material:**

The online version of this article (doi:10.1186/1471-2474-15-425) contains supplementary material, which is available to authorized users.

## Background

OA is a progressive degenerative joint disease caused by wear and tear on the articular surface. OA is thought to be a total joint disease, involving contribution from the articular chondrocytes, sub-chondral bone and synovium [1]. Articular chondrocytes are responsible for producing and maintaining the articular cartilage extracellular matrix that provides a smooth surface for low friction joint movement and “shock absorbing” properties. The synovial cells form a thin lining within the fibrous joint capsule surrounding the joint space. The synovium is composed of synovial fibroblasts and synovial macrophages. The physiologic function of the synovial fibroblasts is to produce a synovial fluid rich in hyaluronan and superficial zone protein/lubricin that lubricates the joint to facilitate low friction movement. While the etiology of OA is complex, biomechanical and biological factors, such as mechanical strain and inflammatory cytokines alter the homeostatic balance between anabolic and catabolic factors in the joint, ultimately leading to the destruction of the articular cartilage. Specifically, synovial cells and articular chondrocytes produce catabolic factors, such as matrix metalloproteinases (e.g., MMP-1 and −13), aggrecanases (e.g., ADAMTS-4 and −5), and pro-inflammatory factors/cytokines (e.g., IL-1, IL-6, TNFα, nitric oxide, prostaglandin E2 (PGE_2_)) that contribute to joint destruction in OA [2–5]. Ultimately, the destruction of articular cartilage can affect joint mobility, leading to severe joint stiffness and pain. Determining the precise contribution and the dynamic interplay between the joint tissue during disease onset and progression is critical to understanding OA and for developing interventions.

One means by which cells and tissues coordinate function is via cell-to-cell communication through gap junction proteins. Gap junctions are made up of connexin monomers that assemble to form a hemichannel. Gap junctions are formed when hemichannels on the plasma membrane of adjacent cells dock to create a transcellular channel. The resultant gap junction channel permits the direct exchange of second messengers, metabolites, ions and other small molecules (<1.0 kDa) among coupled cells. Gap junctions aggregate into large gap junction plaques at the interface of adjacent cells, forming a functional syncytium for the coordinated function of a tissue. Notably, the majority of cells in the joint express the gap junction protein Cx43, including synovial fibroblasts, articular chondrocytes and osteoblasts, as well as cells of the meniscus and ligaments [6–13]. In fact, intercellular gap junctional communication has been demonstrated among chondrocytes of the superficial layer of articular cartilage *in vivo*[14]. *Ex vivo* chondrocytes in cartilage explants have been shown to form functional gap junction networks [15].

In addition to its role in direct gap junctional communication, Cx43 can also form hemichannels that communicate signals directly to the extracellular space [16, 17]. In the cells of bone and cartilage, hemichannels have been implicated in signaling mechanical load responses [18, 19] and are thought to function by serving as conduits for the release of ATP or PGE_2_ into the extracellular milieu following mechanical strain [20]. Regardless of the mode of action (hemichannel or gap junction channel), the relative expression of Cx43 alone impacts signal transduction cascades, gene expression and cell function, at least in bone cells [21].

Several lines of evidence indicate a role for Cx43 in OA. Synovial biopsies from patients with OA have an increase in Cx43 expression and an increase in the size and number of gap junction plaques [22]. Furthermore, *ex vivo* analysis of synovial biopsies of patients with OA revealed that pharmacologic inhibition of Cx43 function reduced the basal and IL-1β-stimulated production of collagenase activity [22, 23]. We have shown that treatment of HIG82 rabbit synoviocytes in culture with IL-1β, a contributor to OA, markedly increases the expression of Cx43 and increased gap junctional intercellular communication among these cells [24]. Similarly, it has been reported that IL-1β enhances Cx43 expression in articular chondrocytes [25, 26]. Further, the density of Cx43 positive cells is markedly enhanced in the superficial zone of osteoarthritic articular cartilage [8]. A more than 40-fold increase in Cx43 protein expression was noted in the articular chondrocytes of OA cartilage compared to healthy controls, with the biggest differences in Cx43 accumulation in the superficial and mid zone of the articular cartilage [13]. High levels of Cx43 staining were seen early in OA and was noted in areas of healthy as well as degraded cartilage, suggesting that altered Cx43 expression may be an early phenotypic change in these cells prior to OA-associated cartilage destruction [13]. However, the mechanism of Cx43 upregulation in OA and the consequence of enhanced Cx43 expression in these cells within the osteoarthritic joint are not yet known. Among osteoblasts, we and others have shown that Cx43 impacts the expression of numerous genes by modulating several signal transduction cascades [21, 27, 28]. In the present study, we examine how increasing Cx43 levels in human and rabbit synovial fibroblasts affect the expression of several OA-associated catabolic and inflammatory genes.

## Methods

### Cell culture and transfection

The HIG82 rabbit synovial fibroblast-like cell line (ATCC) was cultured as described previously [24]. The SW982 human synovial sarcoma cell line (ATCC) was cultured in Leibovitz’s L-15 medium and maintained in a 37°C incubator with atmospheric CO_2_. HIG82 cells were transfected with Lipofectamine 2000 (Life Technologies), as we have published [24]. SW982 cells were transfected with calcium phosphate co-precipitation, as described [29] or with Lipofectamine 2000. The pSFFV-Cx43 construct, which contains the full-length rat Cx43 open reading frame cloned into the EcoR1 site of the pSFFV-neo plasmid [30], was provided by Dr. Thomas Steinberg (Washington University, St Louis, MO). The pSFFV-neo empty vector [31] was provided by Dr. Gabriel Nunez (University of Michigan, Ann Arbor, MI). All plasmid DNA was prepared using PureYield endotoxin free plasmid maxi prep kit (Promega). Non-targeting and human *GJA1* targeting-siRNA Smartpool constructs were purchased from Dharmacon and were used at 25pmol/cm^2^. MG132, dissolved in DMSO, was used at 50 μM. The IKK-2 inhibitor IV (5-(*p*-Fluorophenyl)-2-ureido] thiophene-3-carboxamide) was dissolved in DMSO and used at 10 μM. Cells were treated with MG132, IKK-2 inhibitor IV or DMSO as a vehicle control for 4–5 hours (real time PCR) or 1 hour (immunoblots) prior to harvest. IL-1β was used at 100 ng/ml for 20 minutes to stimulate the phosphorylation of the p65 subunit of NFκB, following pretreatment of the cells with MG132 inhibitor or DMSO control.

### RNA isolation and quantitative RT-PCR

Forty-eight hours post transfection, cells were harvested for RNA extraction using Directzol RNA miniprep (Zymo). RNA (1 μg) was reverse transcribed with either iScript (BioRad) or RevertAid (Fermentas) reverse transcription master mix, according to the manufacturers directions. Quantitative real time PCR was carried out using the SYBR green method, as described previously [24]. For rabbit synovial cell samples, relative gene expression was normalized to 18SrRNA data. For human synovial cell samples, the relative gene expression was simultaneously normalized to the expression of three house keeping genes, *RPL13*, *HPRT* and *GAPDH* using the GeNorm v3.5 Software (Ghent University Hospital Ghent, Belgium), as described previously [32]. All data were also normalized to the expression levels in the empty vector control. The primer sets used for PCR are shown in Table 1.

**Table 1:** **PCR primers**

Target	Species	Primer 1	Primer 2
*MMP1*	Human	TTT GAT GGA CCT GGA GGA AAT C	TGA GCA TCC CCT CCA ATA CC
*MMP13*	Human	ATT AAG GAG CAT GGC GAC TTC T	CCC AGG AGG AAA AGC ATG AG
*ADAMTS4*	Human	CTA TGG GCA CTG TCT CTT AGA CAA AC	CAC TGG CGG TCA GCA TCA
*ADAMTS5*	Human	AAT AAC CCT GCT CCC AGA AAC A	GCG GTA GAT GGC CCT CTT C
*IL1*	Human	CGA ATC TCC GAC CAC CAC TAC	TCC ATG GCC ACA ACA ACT GA
*IL6*	Human	TGT AGC CGC CCC ACA CA	GGA TGT ACC GAA TTT GTT TGT CAA
*PTSG2*	Human	CAG CAC TTC ACG CAT CAG TTT T	CCA GCC CGT TGG TGA AAG
*HPRT*	Human	TGA CAC TGG CAA AAC AAT GCA	GCT TGC GAC CTT GAC CAT CT
*GAPDH*	Human	CCC ACT CCT CCA CCT TTG AC	CAT ACC AGG AAA TGA GCT TGA CAA
*RPL13*	Human	AGC CTT CGC TAG TCT CCG TAT G	TGG CTC TTT TTG CCC GTA TG
*Gja1*	Rat	CGA TTT CCC CGA CGA CAA	TGG CTA ATG GCT GGA GTT CAT
*MMP1*	Rabbit	TGT ATC GTG TTG CAG CTC ATG A	AAA GCC CCA ATA TCA GTA GAA TGG
*MMP13*	Rabbit	AGT AGT TCC AAA GGC TAC AAC TTG TTT	GGA GTG GTC AAG CCC TAA GGA
*ADAMTS4*	Rabbit	ACT GGG TTC CGC GCT ACA	CCT GGC AGG TGA GTT TGC A
*ADAMTS5*	Rabbit	CCT GGG CCC CGA AGA AC	CGA ACG TCA AGT TGC ATT GC
*IL1B*	Rabbit	TCC AGA CGA GGG CAT CCA	TGC CGG AAG CTC TTG TTG TA
*IL6*	Rabbit	GAA CCT GCA GCA GAA AAA CCA	GGC CGC GCA GGA TGA
*PTSG2*	Rabbit	GCT GTG GGC CAG GAA GTG	GCC AGA TTG TGG CAT ACA TCA
*18SrRNA*	multi	CAT TAA ATC AGT TAT GGT TCC TTT GG	TCG GCA TGT ATT AGC TCT AGA ATT ACC

Primer sets for quantitative real time RT-PCR.

### MMP activity assay

MMP activity was measured in conditioned media collected from cultured cells 72 hours post-transfection, using the EnzChek Gelatinase/Collagenase Assay kit (Life Technologies), according to manufacturer’s directions. Briefly, conditioned media (100 μl) was added to the fluorescently tagged gelatin substrate (100 μl, 100 μg/ml final concentration in the supplied 1X reaction buffer). The samples were incubated at room temperature for 2–4 hours and at room temperature, followed by analysis of fluorescent intensity (excitation, 495 nm, emission 515 nm) on a fluorescent microplate reader. Data are displayed relative to the empty vector transfected control.

### Western blotting

Western blotting of whole cell extracts isolated from cells in culture was done as previously described [33]. Briefly, whole cell extracts were prepared from cultured cells 72 hours post transfection using a modified RIPA buffer containing 50 mM Tris, pH 8.0, 150 mM NaCl. 10 mM sodium pyrophosphate, 10 mM sodium fluoride, 10 mM β-glycerophosphate, 1 mM EGTA, 1 mM EDTA, 1% NP-40, 0.5% sodium deoxycholate, 0.1% SDS, and 1X HALT protease and phosphatase inhibitor cocktail (Thermo Scientific). Insoluble material was pelleted, and equal concentrations of the supernatants were electrophoresed on 10% SDS-PAGE gels and transferred to polyvinylidene difluoride membranes. Membranes were blocked in 5% non- fat dry milk, probed with the indicated primary antibodies overnight, and detected with the appropriate horseradish peroxidase–conjugated antibodies and enhanced chemiluminescence detection reagents (BioRad). Blots were acquired and analyzed using an EpiChem gel documentation system (UVP Bioimaging Systems). The rabbit anti-connexin43 antibody was purchased from Sigma. The rabbit anti-phospho-NFκB p65 (Ser536) was purchased from Cell Signaling Technology. The mouse anti-GAPDH antibody was purchased from Millipore. Relative expression was calculated using ImageJ to quantitate band intensity. Data are relative to the expression of GAPDH.

### Statistical analysis

Experiments were repeated a minimum of 3 times with triplicate wells, unless indicated otherwise. Graphs show averages with error bars indicating standard deviations. Samples were compared by an ANOVA for unpaired samples with a Dunnet’s post-hoc test or a t-test, as appropriate, using Prism 6 software. A p-value <0.05 was used as a threshold for statistical significance.

## Results

**Figure 1:**
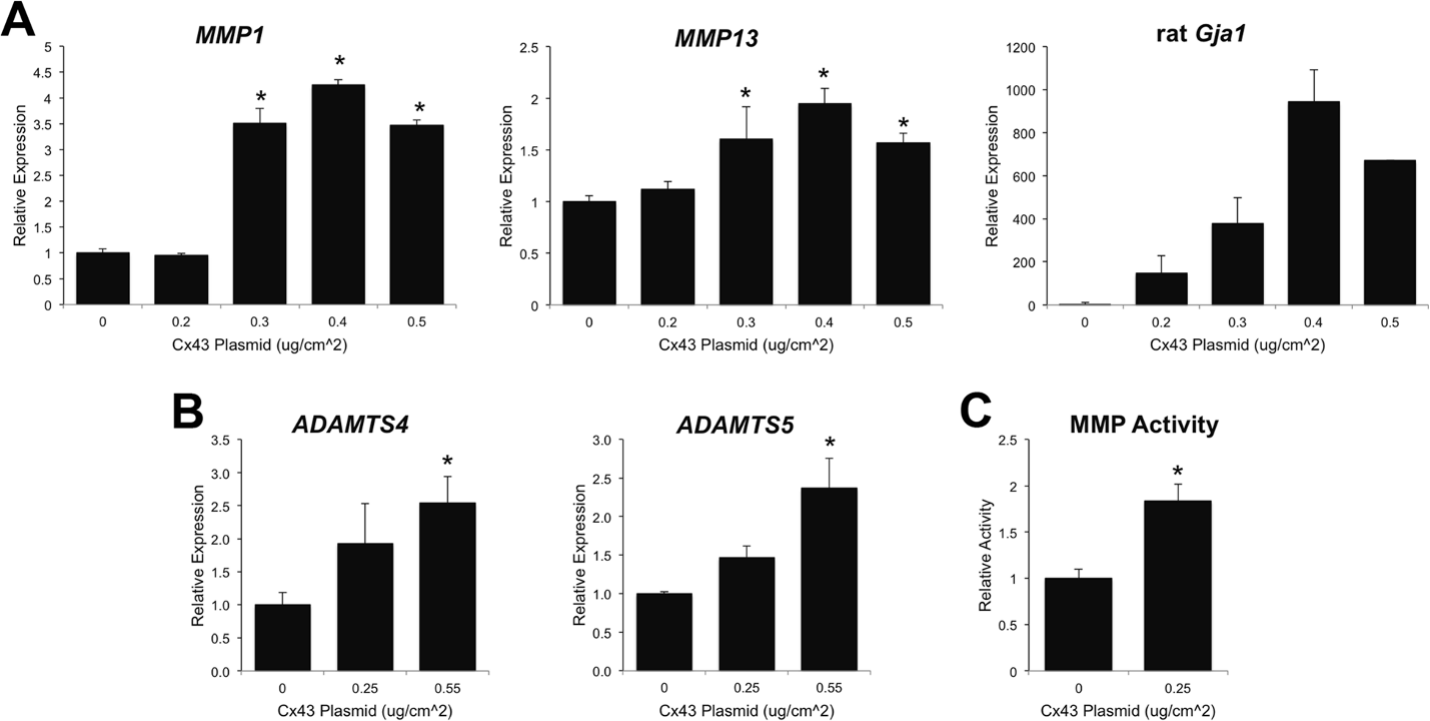
**Transfection with a Cx43 expression construct increased the expression of** ***MMP1*** **,** ***MMP13*** **,** ***ADAMTS4*** **,** ***ADAMTS5*** **mRNA and MMP activity in HIG82 synovial fibroblasts in vitro.** By quantitative real time RT-PCR, transient transfection of HIG82 rabbit synovial fibroblasts with increasing concentrations of a rat Cx43 expressing plasmid (pSFFV-Cx43) dose dependently increased the gene expression of **(A)** *MMP1*, *MMP13* as well as the rat *Gja1* transgene (please note, these primers do not detect the endogenous rabbit Cx43). N = 3. **(B)** Likewise, the gene expression of the aggrecanases, *ADAMTS4* and *ADAMTS5*, were increased by the overexpression of Cx43. N = 3. **(C)** Matrix metalloproteinase activity in conditioned media from Cx43 transfected HIG82 cells was increased nearly 2-fold relative to cells transfected with an empty vector. N = 3. Total DNA was kept constant in all wells by the inclusion of the appropriate amount of an empty vector (pSFFV-neo). Data are shown as means ± standard deviations. Asterisks indicate p-value < 0.05 relative to the empty vector (0 μg) control.

**Figure 2:**
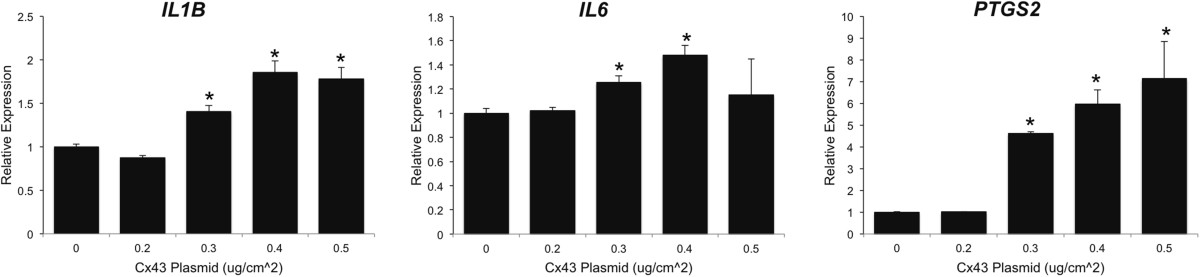
**Transfection with a Cx43 expression construct increased the expression of** ***IL1B*** **,** ***IL6*** **and** ***PTGS2*** **mRNA in HIG82 synovial fibroblasts in vitro.** By quantitative real time RT-PCR, transient transfection of HIG82 rabbit synovial fibroblasts with increasing concentrations of a rat Cx43 expressing plasmid (pSFFV-Cx43) dose dependently increased the gene expression of *IL1B*, *IL6* and *PTGS2*. N = 3. Total DNA was kept constant in all wells by the inclusion of the appropriate amount of an empty vector (pSFFV-neo). Data are shown as means ± standard deviations. Asterisks indicate p-value < 0.05 relative to the empty vector (0 μg) control.

Quantitative real time RT-PCR showed that transient transfection with rat Cx43 (pSFFV-rCx43) in rabbit synovial fibroblasts (HIG82 cells) was sufficient to induce the gene expression of several catabolic factors associated with OA, including the matrix metalloproteinases, *MMP1* and *MMP13*, and the aggrecanases, *ADAMTS4* and *ADAMTS5,* compared to cells transfected with an empty vector (pSFFV-neo) (Figure 1A-B). The expression of these factors dose-dependently increased with the levels of the rat *Gja1* (Cx43) transgene expression. Further, transfection with a Cx43 expressing plasmid in HIG82 cells increased matrix metalloproteinase activity in the conditioned media of these rabbit synovial cells as determined by a fluorometric MMP activity assay (Figure 1C). Similarly, transfection with Cx43 dose-dependently increased the gene expression of the OA-associated inflammatory mediators, *IL1B*, *IL6* and *PTGS2* (COX2) in HIG82 cells (Figure 2).

Next, we wanted to show that disruption of Cx43 expression or function would reduce expression of these genes. However, attempts to knockdown Cx43 expression with siRNAs were unsuccessful in these rabbit synovial cells. Meanwhile, pharmacologic inhibition of gap junction function with carbenoxolone or heptanol was effective at reducing gene expression and matrix metalloproteinase production in HIG82 cells, but issues of cell viability/cell health in the presence of these inhibitors made interpretation difficult (data not shown). As a result, we instead examined the effects of Cx43 on the function of human synovial fibroblast cell line (SW982) in which we could effectively knockdown Cx43 gene expression with commercial siRNAs. SW982 cells are derived from a human synovial sarcoma and behave similarly to human synovial fibroblasts including their ability to produce inflammatory and catabolic factors, including IL-1, IL-6, Cox2/*PTGS2*, iNOS/*NOS2*, TGFβ, MMP-1, −2, −3 and −13 and ADAMTS-4, both basally and at markedly increased levels following treatment with IL-1β [34, 35].

**Figure 3:**
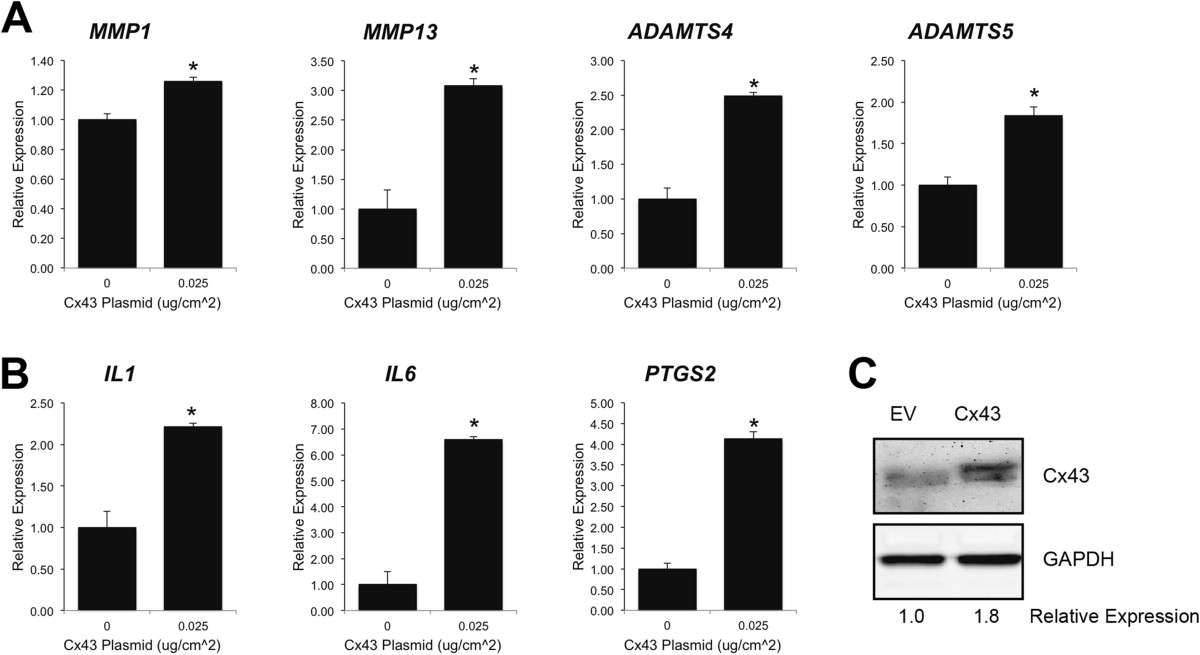
**Transfection of human synovial fibroblast-like cells with a Cx43 expression construct increased the expression of OA-associated catabolic and inflammatory genes.** By quantitative real time RT-PCR, transient transfection of SW982 human synovial fibroblast-like cells with a rat Cx43 expressing plasmid (pSFFV-Cx43) significantly increased the gene expression of the OA-associated catabolic genes **(A)** *MMP1*, *MMP13*, *ADAMTS4* and *ADAMTS5* and the inflammatory cytokine genes **(B)** *IL1*, *IL6* and *PTGS2*. N = 3. Data are shown as means ± standard deviations. Asterisks indicate p-value < 0.05 relative to the empty vector control. **(C)** Immunoblotting of transfected SW982 cells showed an increase in Cx43 expression in pSFFV-Cx43 transfected cells relative to cells transfected with an empty vector (EV). GAPDH was used as a load control.

**Figure 4:**
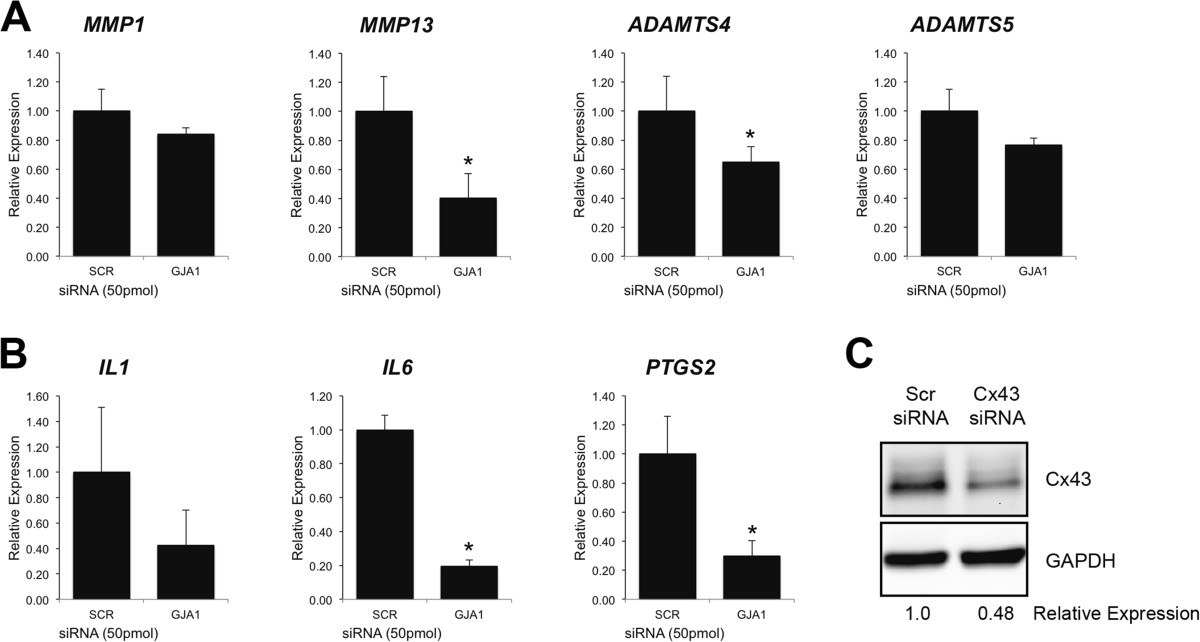
**Cx43 Knockdown in human synovial fibroblast-like cells reduced the expression of OA-associated catabolic and inflammatory genes.** By quantitative real time RT-PCR, transient transfection of SW982 human synovial fibroblast-like cells with *GJA1* targeting siRNAs (*GJA1*) reduced the gene expression of some OA-associated **(A)** catabolic genes and **(B)** inflammatory genes relative to a scrambled siRNA control (SCR) N = 3. Data are shown as means ± standard deviations. Asterisks indicate p-value < 0.05 relative to the empty vector control. **(C)** Immunoblotting of transfected SW982 cells confirmed a decrease in Cx43 expression in *GJA1* siRNA transfected cells relative to cells transfected with a scrambled control siRNA (SCR). GAPDH was used as a load control.

As was noted for HIG82 cells, increasing Cx43 protein expression (Cx43 protein levels were typically 1.5-2.5 fold higher than empty vector transfected controls as determined by western blotting) in SW982 human synovial cells by transient transfection enhanced the gene expression of *MMP1*, *MMP13*, *ADAMTS4*, *ADAMTS5*, *IL1*, *IL6* and *PTGS2* compared to cells transfected with an empty vector (Figure 3). Conversely, when Cx43 expression was knocked down in SW982 cells with siRNA directed against *GJA1* (the gene encoding Cx43), the gene expression of *MMP13*, *ADAMTS4*, *IL6* and *PTGS2* were significantly reduced relative to cells transfected with a scrambled siRNA control (Figure 4A). The gene expression of *MMP1*, *ADAMTS5* and *IL1* were also mildly reduced but did not reach statistical significance.

**Figure 5:**
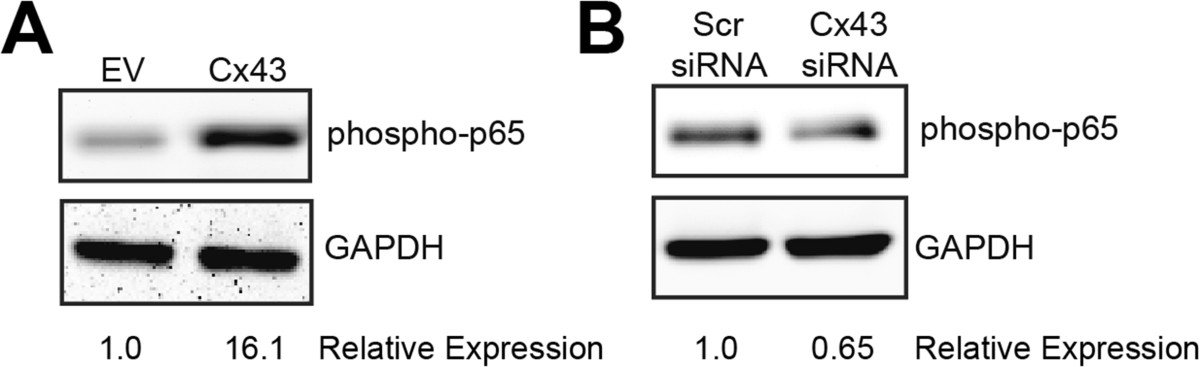
**Modulation of Cx43 expression affected the NFκB pathway.** Immunoblots from whole cell extracts of transiently transfected SW982 cells showed that **(A)** overexpression of Cx43 enhanced the abundance of phospho-p65 (RelA) subunit of the NFκB complex while **(B)** knockdown of Cx43 expression had the opposing effect. GAPDH was used as a load control.

**Figure 6:**
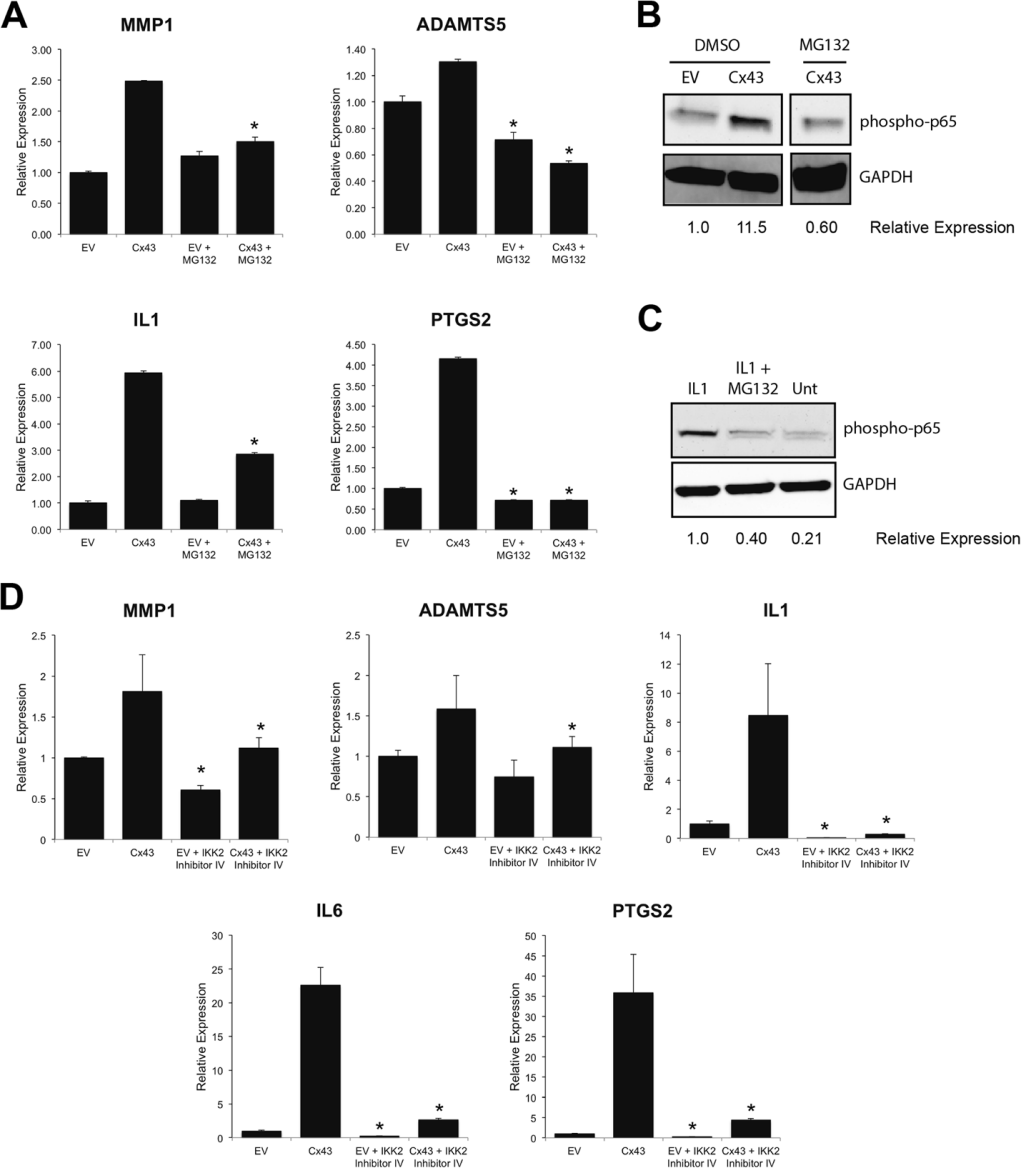
**Inhibition of the NFκB pathway prevented the ability of Cx43 overexpression to enhance the expression of some OA-associated catabolic and inflammatory genes.** By quantitative real time RT-PCR, transient transfection of SW982 human synovial fibroblast-like cells with a rat Cx43 expressing plasmid (pSFFV-Cx43) significantly increased the gene expression of the OA-associated catabolic and inflammatory genes **(A)** *MMP1*, *ADAMTS5, IL1* and *PTGS2*. This effect of Cx43 on the expression of these genes was abrogated by inhibition of the NFκB pathway with MG132 (50 μM, 5 hours). N = 3. Data are shown as means ± standard deviations. Asterisks indicate p-value < 0.05 relative to the corresponding vehicle treated control. **(B)** Immunoblots showed that Cx43 overexpression increased the abundance of phospho-p65 NFκB, an effect that could be inhibited by exposure to MG132 (50 μM, 1 hours). GAPDH was used as a load control. This blot is from a single gel and single exposure but is from non-contiguous lanes. The irrelevant lanes have been digitally removed. **(C)** Immunoblot with anti-phospho-p65 NFκB antibodies confirms the effectiveness of MG132 to inhibit the NFκB pathway when the pathway is stimulated by IL1β (100 ng/ml, 20 minutes). **(D)** Treatment with the NFκB pathway inhibitor (IKK2-inhibitor IV, 10 μM, 4 hours) reduced the Cx43-dependent expression of several OA-associated genes, as determined by quantitative real time RT-PCR. N = 3. Data are shown as means ± standard deviations. Asterisks indicate p-value < 0.05 relative to the corresponding vehicle treated control.

Next, we examined the underlying molecular mechanisms by which Cx43 may impact expression of the genes. Because many of the Cx43-sensitive genes that we tested are direct targets of the canonical NFκB cascade, we evaluated the influence of Cx43 on this pathway. Examination of the phosphorylation of the p65 subunit of NFκB by western blotting revealed that increasing Cx43 expression enhanced phospho-p65 levels, while Cx43 knockdown diminished phospho-p65 levels (Figure 5). Importantly, inhibition of the NFκB pathway with MG132 blocked the impact of Cx43 overexpression on the production of a subset of these OA-associated catabolic and inflammatory genes, including *MMP1*, *ADAMTS5*, *IL1* and *PTGS2* and reduced the amount of phospho-p65 in Cx43-expressing SW982 cells (Figure 6A-C). Similarly, use of the NFκB pathway inhibitor, IKK-2 inhibitor IV, blocked the effects of Cx43 overexpression of several OA-associated genes (Figure 6D).

## Discussion

In this manuscript, we showed that increasing Cx43 levels in synovial cells is sufficient to enhance the expression of OA-associated catabolic and inflammatory genes in both rabbit and human synovial fibroblasts. Given that Cx43 is upregulated in both articular chondrocytes and synovial cells in OA [13, 22], as well as the present data and the data of others suggesting that Cx43 can influence the expression of genes associated with OA [22, 23], it is important to consider that Cx43 could contribute to the subsequent pathology of the joint and may be a therapeutic target to treat OA. Of course, it must be acknowledged that this work is done in cell lines and needs to be confirmed in vivo. Promisingly, in a rat model of collagen induced rheumatoid arthritis, silencing Cx43 expression with siRNA diminished both inflammation and ankle joint destruction [36]. In addition, *ex vivo* Cx43 siRNA reduced the expression of inflammatory cytokines in rat fibroblast-like synovial cells from these animals [36]. These findings are consistent with our data, which also show that knockdown of Cx43 can reduce the expression of a subset of OA-associated catabolic genes and inflammatory genes. In fact, in our systems all of the tested OA-associated genes were diminished by transfection of the cells with *GJA1*-targeted siRNAs, even if only a subset reached statistical significance. Importantly, our model of Cx43 overexpression consistently produces a 1.5-2 fold increase in Cx43 protein in the human synoviocytes. This is consistent with the level of expression observed in synovial biopsies from OA patients relative to non-OA patients [22] and substantially less than the ~30-fold increase in Cx43 protein observed in articular chondrocytes from patients with OA [13].

Little is known regarding the molecular mechanisms by which alterations of Cx43 could affect gene expression in synovial cells. Our previous work in bone cells has shown that numerous signal transduction cascades, such as ERK and Protein Kinase C delta (PKCδ), can influence gene expression downstream of Cx43 [32, 33, 37]. Others have shown in bone cells that Cx43 can regulate other pathways, including β-catenin expression [38, 39] and βarrestin/cAMP signaling [40]. Less is known regarding the role of Cx43 and downstream signaling in the synovium or articular cartilage, beyond the propagation of Ca^2+^ waves [41, 42]. In the present study, we showed that Cx43 regulates the NFκB signaling pathway in synovial fibroblasts, with gain of Cx43 increasing and loss of Cx43 decreasing signaling through this pathway. It is not surprising that NFκB is a target influencing the expression of a subset of these OA-associated genes, as this pathway is intimately associated with OA and has been a target of therapeutic intervention in arthritic disease [43, 44]. Rather, what is novel is that this is the first study that we are aware of that shows that Cx43 regulated the expression of OA-associated genes and the activity of the NFκB pathway. Our western blotting data clearly showed modulation of phosphorylation of p65 RelA as a result of increasing or decreasing Cx43 in these cells. Further, two pharmacologic inhibitors of the NFκB pathway markedly reduced the effect of Cx43 on gene expression. Of course, studying NFκB pathway signaling in the context of a synovial sarcoma cell line (SW982) is a limitation as altered NFκB signaling is a hallmark of these cells. However, we have observed a similar regulation of NFκB signaling by Cx43 in other cell types as well (data not shown). Even if these synovial sarcoma cells have elevated basal NFκB, there is little reason to suspect that the mechanisms by which Cx43 modulates this pathway are not conserved among primary cells. Nonetheless, future experiments will examine the regulation of these synovial cell function in primary cells in culture or *in vivo*. Importantly, as has been observed in osteoblasts and osteocytes, it is unlikely that NFκB is the only signaling pathway affected downstream of Cx43 in synovial cells. Indeed, Cx43 interacts with numerous signaling molecules and can affect signaling by modulating cell-to-cell diffusion of second messengers, as well as by structural interactions [21, 45–47]. Future studies will look at signal transduction cascades such as PKCδ, which is involved in Cx43-dependent signaling in bone cells where it converges on Runx2 [48] and has also been implicated in OA pathogenesis and the expression of *MMP13* via Runx2 [49, 50].

Importantly, we do not know if this same Cx43-dependent effect is also present in articular chondrocytes. While numerous studies have shown that Cx43 is expressed in articular chondrocytes, how Cx43 functions in these cells is uncertain. Cx43 can function as unopposed hemichannels in articular cartilage [8, 51, 52], and the mechanisms that govern signal transduction in the context of classic cell-to-cell communication through gap junctions are likely different than with hemichannels [53]. In addition, studies have shown functional cell-to-cell communication via classic gap junctions between articular chondrocytes in the superficial layer, as well as extensive gap junctional coupling among articular chondrocytes in a network that is analogous to the osteocytic canalicular network found in bone [13–15]. Future studies will investigate the role of Cx43 in articular chondrocyte function.

Interestingly, this is the first study to show that Cx43 abundance can impact the expression of *PTGS2*, the gene encoding COX2, an enzyme involved in the production of PGE_2_. Studies have shown a relationship between Cx43 and PGE_2_, particularly in response to mechanical load in osteocytes and articular chondrocytes, with mechanical stimulation increasing Cx43 expression and subsequently PGE_2_ release [52, 54, 55]. Those studies have focused to some extent on the Cx43-dependent release of PGE_2_ from cells. If Cx43 can also influence the transcription of *PTGS2* and the synthesis of PGE_2_ in those systems, as it does in synovial cells, it should also be considered that the increase in PGE_2_ observed in osteocytes and articular chondrocytes following mechanical stimulation may be secondary to or in addition to the transcriptional control of *PTGS2* by Cx43. Future studies will need to clarify this issue.

Combined with our previous work and the work of others showing increases in Cx43 in synovial cells the OA joint, the present data support a model in which OA-associated changes in the joint (e.g., inflammation, altered mechanical load) can increase Cx43 expression. Further, Cx43 upregulation is sufficient to lead to the production of additional catabolic and inflammatory factors. The result is a vicious cycle that culminates in joint destruction. Accordingly, it is reasonable to predict that targeting Cx43 or at least the subset of signal transduction cascades affected by Cx43 abundance could offer therapeutic benefit in the treatment of OA.

## Conclusions

Increased levels of Cx43 are observed in synovial biopsies from patients with OA. Accordingly, we examined the impact of increased Cx43 on the expression of OA-associated genes. Increasing the levels of Cx43 is sufficient to drive the expression of catabolic and inflammatory genes by synovial cells, while knockdown of Cx43 could decrease the expression of these genes. The NFκB cascade mediates the effect of Cx43 on the expression of a subset of these OA-associated genes. As such, targeting Cx43 expression or function may be a viable therapeutic strategy to attenuate the catabolic and inflammatory environment of the joint during OA.

## Authors’ original submitted files for images

Below are the links to the authors’ original submitted files for images. Authors’ original file for figure 1 Authors’ original file for figure 2 Authors’ original file for figure 3 Authors’ original file for figure 4 Authors’ original file for figure 5 Authors’ original file for figure 6

## Abbreviations

ADAMTS4: Aggrecanase-1
ADAMTS5: Aggrecanase-2
Cx43: Connexin43
IL: Interleukin
MMP: Matrix metalloproteinase
OA: Osteoarthritis
RT-PCR: Reverse transcription-polymerase chain reaction.
